# Significance of 4D US parameters for the clinical treatment of female patients with stress urinary incontinence

**DOI:** 10.3389/fsurg.2023.1126293

**Published:** 2023-07-21

**Authors:** Changqin Jiang, Song Zhang, Jing Chen, Yangyang Zhang, Keke Cai, Wei Chen, Yuanyuan Wu, Chaozhao Liang

**Affiliations:** ^1^ Department of Urology, The First Affiliated Hospital of Anhui Medical University, Hefei, China; ^2^ The Institute of Urology, Anhui Medical University, Hefei, China; ^3^ Anhui Province Key Laboratory of Genitourinary Diseases, Anhui Medical University, Hefei, China; ^4^ Department of Ultrasound, The First Affiliated Hospital of Anhui Medical University, Hefei, China

**Keywords:** 4D pelvic floor ultrasound, transobturator suburethral tape (TOT), stress urinary incontinence (SUI), postoperative complication, female patients

## Abstract

**Background:**

Stress urinary incontinence (SUI) that has been associated with abnormal pelvic floor muscle function or morphology is a common condition. This research aimed to study the impact of the four-dimensional (4D) pelvic floor ultrasound on the treatment of female patients with clinical diagnosis of SUI and to evaluate its clinical significance on SUI.

**Methods:**

We enrolled 51 women with SUI. Before transobturator suburethral tape procedures, the patients underwent 4D pelvic floor ultrasonography. The measurements include residual urine volume, bladder detrusor thickness in resting state, the vertical distance from the bladder neck to the posterior inferior edge of pubic symphysis at rest and Valsalva movement, posterior angle of bladder urethra, and urethral rotation angle. The degree of movement of the bladder neck (the difference between the vertical distance from the bladder neck to the posterior inferior edge of the pubic symphysis under the resting state and the maximum Valsalva movement) and the formation of a funnel at the internal orifice of the urethra were calculated.

**Results:**

The mean bladder detrusor thickness was 2.6 ± 0.9 mm, the vertical distance from the bladder neck to the posterior inferior edge of pubic symphysis was 27.7 ± 4.5 mm, the posterior angle of the bladder was 122.7 ± 18.9°, the vertical distance from the rectal ampulla to the posterior inferior edge of pubic symphysis was 18.5 ± 4.6 mm, and the mean area of hiatus of the levator ani muscle was 22.1 ± 6.0 cm^2^. The mean posterior angle of the bladder on Valsalva was 159.3 ± 23.1°, and the mean urethral rotation angle was 67.2 ± 21.4°.

**Conclusions:**

The 4D pelvic floor ultrasound is a reliable method in evaluating preoperational morphological characteristics of patients with SUI. With the help of the 4D pelvic floor ultrasound, the individualized treatment regimen can be developed and, more importantly, the inappropriate surgical decision can be avoided.

## Introduction

Urinary incontinence was defined as a social or hygienic problem by the International Continence Society due to the involuntary loss of urine (1). Stress urinary incontinence (SUI) as a common type of urinary incontinence, involuntary urinary leakage during exercise or coughing or laughing, is often underestimated and undertreated (2, 3). According to surveys, the prevalence of urinary incontinence is about 35% among women over the age of 18 (4). As the population aging continues to intensify, the prevalence of urinary incontinence and the demand for the treatment of SUI will continue to increase (5). Surgery is the ultimate option for the treatment of SUI with high cure rates. Mid-urethral slings, mini-slings, and bulking agents have commonly used minimally invasive procedures for the treatment of SUI (6, 7). Transobturator suburethral tape (TOT) is one of the important methods for the surgical management of SUI with high cure rates (8, 9).

Some studies showed that SUI could be accompanied by the loss of the posterior urethrovesical angle during the increasing of intra-abdominal pressure (10) and deficiency of urethral sphincter function, characterized by an open vesical neck and proximal urethra at rest with minimal or no urethral descent during stress, maybe one of the important reasons the mid-urethral slings operation failed for some patients (11).

Four-dimensional (4D) pelvic floor ultrasound is a safe and non-invasive tool, which has been widely used as a tool to virtually reconstruct the anatomical structure of the female urethra in SUI (12–14). In previous literature, measuring the ultrasonic parameters of the tape at a single point or multiple positions has been used to explore the mechanism of TOT procedures, including morphological information changes and the anatomical morphology, function, and behavior of the surgical site (13–18).

However, there is still a lack of standardized methods to comprehensively evaluate the preoperative pelvic floor morphology of patients who are to undergo TOT procedures. Considering that there are differences in the appearance of the pelvic floor during increased intra-abdominal pressure compared with the resting state, that difference may be related to the incidence of complications, such as urinary retention (voiding dysfunction >24 h) or the unsatisfactory improvement of urinary leakage symptoms after operation.

Therefore, we proposed a hypothesis that different preoperative pelvic floor morphology in female patients with SUI could reflect different dynamics and functions, and for patients with SUI, the individualized treatment regimen can be developed and inappropriate surgical decision can be avoided by the preoperative parameters measurement of the pelvic floor by the 4D pelvic floor ultrasound. Accordingly, we designed this study to clarify the significance of the 4D pelvic floor ultrasound for the clinical treatment of female patients with SUI.

## Methods and instruments

**Figure 1:**
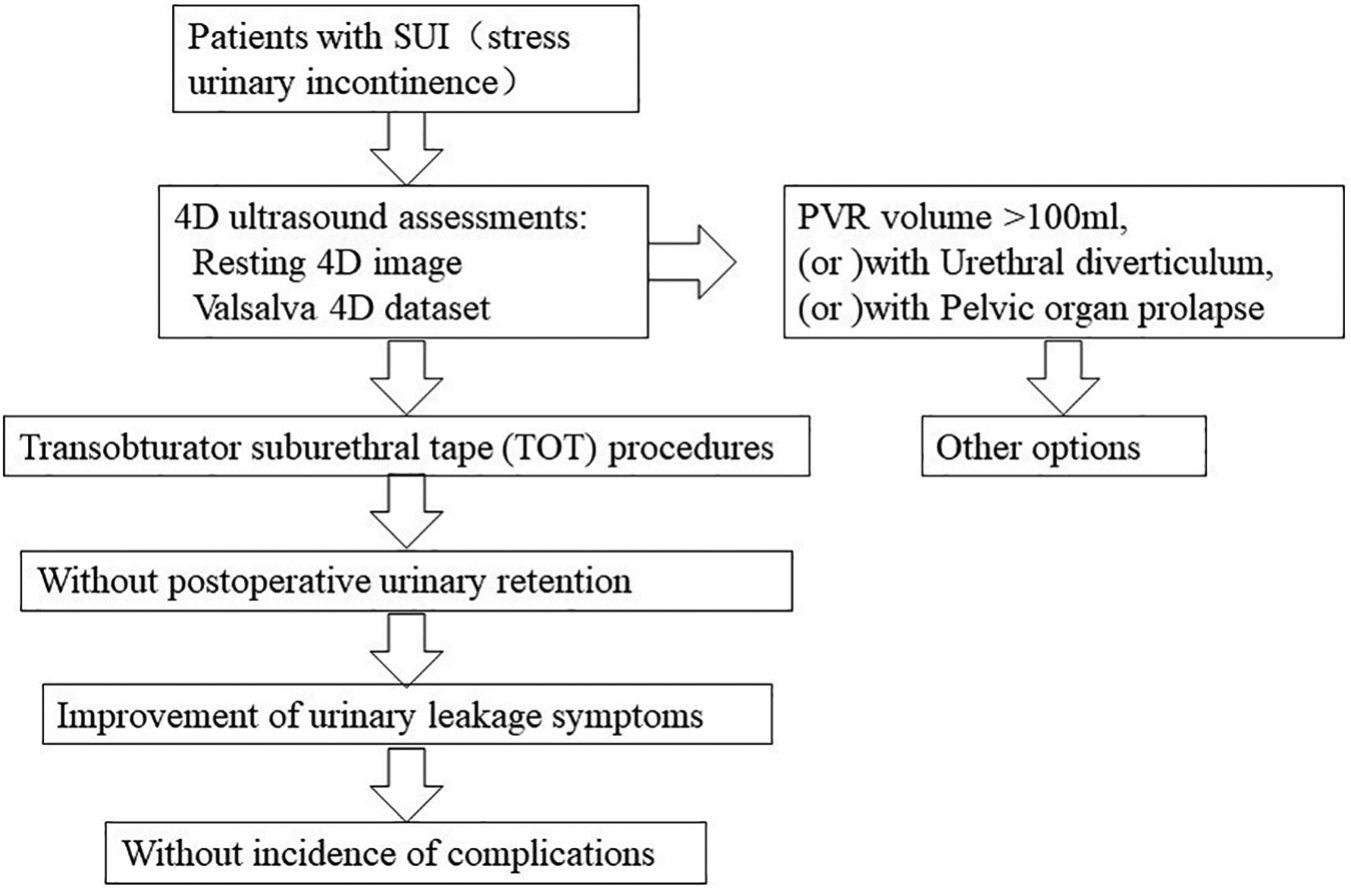
After the patients with SUI preoperative 4D pelvic floor ultrasound assessments, excluding PVR volume greater than 100 ml, or urethral diverticulum, or pelvic organ prolapse, the rest of the patients underwent TOT procedures.

Between May 2020 and October 2020, we retrospectively reviewed the 4D pelvic floor ultrasound data sets of 58 women who underwent TOT surgery for SUI. All the 4D pelvic floor ultrasound data sets of the patients were provided by two experienced investigators in The First Affiliated Hospital of Anhui Medical University. After excluding the patients with histories of diabetes, neurologic diseases, stroke, mixed urinary incontinence, cystocele or uterine prolapse, vascular disease, obesity with metabolic syndrome, previous vaginal surgery due to prolapse or urinary incontinence, or incomplete data from ultrasound assessments, the 4D pelvic floor ultrasound data sets of 51 women were recruited for analysis (Figure 1). Informed consent was provided by all the participants. The methods, definitions, and units conform to the standards recommended by the International Continence Society (19), except where specifically noted. Before the examination, the patients emptied the rectum and the bladder and lie down in the lithotomy position. After the probe was placed in the perineum with real-time four-dimensional mode, the volume data of the median sagittal plane under the resting state and the movement with maximum intra-abdominal pressure (Valsalva, the patient was asked to hold her breath and exerted downward) was collected, and the changes of the 4D pelvic floor ultrasound images in the two states were observed. The measurements include residual urine volume, bladder detrusor thickness in resting state, vertical distance from the bladder neck to the posterior inferior edge of pubic symphysis at rest and Valsalva movement, posterior angle of bladder urethra (angle between the proximal urethra and outer wall of bladder triangle), and urethral rotation angle (angle between the proximal urethral axis and the middle axis of the human body). The degree of movement of the bladder neck (the difference between the vertical distance from the bladder neck to the posterior inferior edge of the pubic symphysis under the resting state and the maximum Valsalva movement) and the formation of a funnel at the internal orifice of the urethra were calculated (20).

## Statistical analysis

The data were presented as mean ± standard deviation. All analyses were carried out using SPSS for Windows (version 23.0) software (SPSS, Inc., Chicago, IL, United States).

## Results

The 4D pelvic floor ultrasound data sets of 51 women were included for analysis. With the comprehensive evaluation of the preoperative pelvic floor morphology, none of the 51 patients in our study suffer from infection, hematoma, pain, or urinary retention. Of the 51 women, the mean age was 54 ± 7 years; the median parity was 1, and the mean body mass index was 24.2 ± 2.7 kg/m^2^. Of the 51 women, 28 (54.9%) were postmenopausal, and 7 (13.7%) had previously undergone hysterectomy. Besides, 9 of the 51 patients (17.6%) had post-void residual (PVR, the urine that remains in the bladder after voiding), but none of the PVR volumes were more than 100 ml. The general characteristics are summarized in Table 1.

**Table 1:** General characteristics of the participants.

Demographic and clinical data of the study group (*N* = 51)	
Age (SD) (years)	54 ± 7
Parity median (range)	1 (0–4)
BMI (SD) (kg/m^2^)	24.2 ± 2.7
Nulliparous women, *n* (%)	1 (2.0%)
Women with PVR, *n* (%)	9 (17.6%)

PVR is the urine that remains in the bladder after voiding.

All of them received preoperative 4D pelvic floor ultrasound assessments at rest and during Valsalva movement. A total of 14 out of 51 patients (27.5%) had suffered the formation of a funnel at the internal orifice of the urethra. One of the patients (2.0%) may have experienced dysuria, whose residual urine volume was more than 50 ml. The results of bladder detrusor thickness, the descent of the rectal ampulla relative to the symphysis pubis at rest, and the posterior angle of the bladder are summarized in Table 2. The mean bladder detrusor thickness was 2.6 ± 0.9 mm, the vertical distance from the bladder neck to the posterior inferior edge of pubic symphysis was 27.7 ± 4.5 mm, the posterior angle of the bladder was 122.7 ± 18.9°, the vertical distance from the rectal ampulla to the posterior inferior edge of pubic symphysis was 18.5 ± 4.6 mm, and the mean area of hiatus of the levator ani muscle was 22.1 ± 6.0 cm^2^. The preoperative ultrasound measurements during the Valsalva movement are summarized in Table 3. The mean posterior angle of the bladder on Valsalva was 159.3 ± 23.1°, and the mean urethral rotation angle was 67.2 ± 21.4°.

**Table 2:** The 4D pelvic floor ultrasound data sets of the patients at rest.

Preoperative ultrasound measurements at rest (*N* = 51)	
Bladder detrusor thickness (SD) (mm)	2.6 ± 0.9
The vertical distance from the bladder neck to the posterior inferior edge of the pubic symphysis (SD) (mm)	27.7 ± 4.5
The posterior angle of the bladder (SD) (°)	122.7 ± 18.9
The vertical distance from the rectal ampulla to the posterior inferior edge of the pubic symphysis (SD) (mm)	18.5 ± 4.6
Area of hiatus of levator ani muscle (SD) (cm^2^)	22.1 ± 6.0

**Table 3:** The 4D pelvic floor ultrasound data sets of the patients during Valsalva movement.

Preoperative ultrasound measurements during Valsalva movement (*N* = 51)	
The posterior angle of the bladder (SD) (°)	159.3 ± 23.1
Urethral rotation angle (SD) (°)	67.2 ± 21.4

Therefore, the goal of this study was to demonstrate the significance of the 4D pelvic floor ultrasound for the clinical treatment of female patients with SUI. With the help of the 4D pelvic floor ultrasound, the individualized treatment regimen can be developed and, more importantly, the inappropriate surgical decision can be avoided.

## Discussion

Urinary incontinence was defined as a social or hygienic problem by the International Continence Society due to the involuntary loss of urine (1), and SUI is a common type of urinary incontinence among women of middle age and elderly age. There are many factors related to the occurrence and progression of SUI. Besides the main risk factors such as advanced age and pregnancy and delivery, two of them are paid much attention in recent studies: the fascial structures of urethral support, which govern urethral mobility, and the urethral sphincter complex (USC), which provides the urethral tension (6). According to general recognition, the urethral closure pressure is the key factor in maintaining continence. With the increase of the abdominal pressure, the urethral closure pressure will be lower than the intravesical pressure, resulting in an unconscious urine leakage. The lack of urethral closure pressure is associated with anatomic changes in the bladder and urethra (21, 22). After the failure of conservative treatment, surgical treatment has become the priority treatment for the purpose to reconstruct the anatomical structure and function (23). TOT procedure is one of the effective options for SUI. The preoperative pelvic floor morphology of patients by the 4D pelvic floor ultrasound can help with assessing whether the urethral funnel is formatted, which is one of the contraindications to the surgery (12). The early complications of TOT surgery, including voiding dysfunction, pain, or the unsatisfactory improvement of urinary leakage symptoms, result in a poor subjective evaluation of urinary symptoms following the operation. A study of 446 patients suggested that the incidence of early postoperative complications was as follows: persistent SUI (0.7%) and voiding dysfunction >24 h (10.3%) (24).

The patients in our study emptied both the rectum and bladder before the examination, while some other studies had acquired dynamic data sets imaging with a bladder volume of 200–300 ml (25), or data sets with no more than 200 ml urine in the bladder (26). This may result in a lack of pelvic floor morphological data at the leakage point. A urodynamic evaluation, which is used to distinguish the type of urinary incontinence in female patients and facilitates doctors to choose the more appropriate treatment (27, 28), can detect leakage point pressure and depict pelvic floor morphological at leakage point combining with ultrasound. Urodynamics (UDS) testing has some requirements for the patients. For example, the patients are always required to have a natural urine output of over 150 ml to ensure the feasibility of the urodynamic examination results. However, some patients are still unable to meet this requirement. The preoperative pelvic floor morphology will be more accurately and comprehensively measured for the 4D pelvic floor ultrasound data of the patients with SUI filling by larger sample and UDS data. Furthermore, the 4D pelvic floor ultrasound may replace UDS as one of the primary tests for preoperative evaluation of the patients with urine incontinence.

The current study may be limited because control group data was not collected (15, 29, 30). Besides, all the stored 4D pelvic floor ultrasound data sets are stable and all the examinations lack duplication, so the analysis of these data sets might not truly be reflective of the clinical practice and the achieved perfect consistency (25).

## Conclusion

The 4D pelvic floor ultrasound could offer a reliable method in evaluating preoperational morphological characteristics of the patients with SUI. These data sets could facilitate doctors to understand the anatomical morphology characteristics of the pelvic floor of the patients. With the help of the 4D pelvic floor ultrasound, the individualized treatment regimen can be developed and, more importantly, the inappropriate surgical decision can be avoided.

## Data Availability

The raw data supporting the conclusions of this article will be made available by the authors, without undue reservation.
